# Evaluation of the effects of DBS in the caudal Zona incerta on brain activity during a working memory task in patients with essential tremor

**DOI:** 10.1016/j.ynirp.2023.100193

**Published:** 2023-11-01

**Authors:** Johanna Philipson, Amar Awad, Lena Lindström, Patric Blomstedt, Marjan Jahanshahi, Johan Eriksson

**Affiliations:** a Department of Clinical Sciences, Neuroscience, Umeå University, Sweden; b Umeå Center for Functional Brain Imaging (UFBI), Dept. of Integrative Medical Biology, Umeå University, Sweden; c Department of Psychology, Lund University, Lund, Sweden; d Unit of Functional Neurosurgery, UCL Queen Square Institute of Neurology, London, UK

**Keywords:** Deep brain stimulation, Essential tremor, Working memory, fMRI

## Abstract

Essential tremor (ET) is characterized by bilateral upper limb postural and/or kinetic tremor, but also cognitive deficits. Tremor in ET, as well as aspects of cognitive deficits associated with ET, have been suggested to be linked to dysfunction in the cerebello-thalamo-cerebral circuit. In ET patients with disabling and medically intractable motor symptoms, Deep Brain Stimulation (DBS) is effective in reducing tremor. DBS in the caudal Zona incerta (cZi) has been shown to modulate the activity of the sensorimotor cerebello-cerebral circuit during motor tasks. Whether the activity in the cerebello-cerebral circuit is modulated by DBS during tasks involving working memory is unknown. The present study therefore aimed to investigate the possible effects of cZi DBS on working-memory processing in ET patients by means of task-based blood oxygen level-dependent (BOLD) fMRI.

Thirteen ET patients completed a working-memory task during DBS OFF and ON conditions. The task involved three conditions: maintenance, manipulation, and control. Behaviorally, there was no significant effect from DBS on accuracy, but a marginally significant Task x DBS interaction was detected for response times (RTs). However, post hoc comparisons for each condition failed to reach statistical significance. FMRI analyses revealed that DBS did not alter BOLD signal in regions of interest (lateral prefrontal cortex, parietal cortex, and the cerebellum), or in a complementary whole-brain analysis.

The present study indicates that DBS in the cZi in patients with ET has at most marginal effects on working memory, which is consistent with the results of pre- and post-DBS neuropsychological assessment showing minimal cognitive effects of surgery.

## 1 Introduction

Essential tremor (ET) is the most common movement disorder in adults. It is a progressive neurological disease, primarily causing motor symptoms characterized by bilateral upper limb postural and/or kinetic tremor (Louis and Ferreira, 2010). Although the etiology of ET has still not been identified, the current view on ET symptomatology has expanded from a monosymptomatic clinical phenotype, to include a broader description of both motor and non-motor features. Non-motor features in ET include cognitive deficits, sleep abnormalities, and psychiatric symptoms such as depression and anxiety (Chandran and Pal, 2012). Cognitive deficits related to ET have primarily been found within the domains of executive function and attention (Gasparini et al., 2001; Higginson et al., 2008; Lombardi et al., 2001; Passamonti et al., 2011; Tröster et al., 2002), and have been suggested to be associated with a dysfunction in the cerebello-thalamo-cerebral network. For example, ET patients exhibit abnormally enhanced cerebellar responses during tremor-inducing motor tasks and during high-load working-memory tasks (Passamonti et al., 2011; Broersma et al., 2016). Other studies have suggested that not only the cerebello-thalamo-cerebral network is involved, but also several other brain structures and pathways as well as atrophy of grey matter volume in the frontal parietal lobes, cingulate and insular cortices and in the cerebellum posterior lobe (Bhalsing et al., 2014; Sengul et al., 2022). This would suggest that cognitive deficits in ET are not limited to a fronto-cerebellar or dysexecutive syndrome, but also involve structures outside these networks.

In ET patients with disabling and medically intractable symptoms, Deep Brain Stimulation (DBS) is effective in reducing tremor (Deuschl et al., 2011). Traditionally, surgery for tremor has targeted the ventral intermediate nucleus (Vim) of the ventrolateral thalamus (Hopfner and Deuschl, 2020). However, targeting the caudal Zona incerta (cZi), within the posterior subthalamic area (PSA) has been shown to be more effective and more efficient than Vim-DBS in alleviating tremor (Deuschl et al., 2011; Barbe et al., 2018; Blomstedt et al., 2010; Fytagoridis et al., 2012; Plaha et al., 2011; Sandvik et al., 2012; Kvernmo et al., 2022)

The cognitive side-effects of Vim-DBS are well known and have been investigated in several studies, primarily revealing a decline in verbal fluency and, in a recent study, verbal memory (Dhima et al., 2021; Ehlen et al., 2017; Fields et al., 2003; Lucas et al., 2000; Tröster et al., 1999). In addition, there have also been reports of absence of cognitive change related to DBS in Vim (Heber et al., 2013; Klein et al., 2017). There is, however, paucity of data on the possible effects on cognition in cZi-DBS. Given the cognitive deficits in ET patients (Chandran and Pal, 2012; Janicki et al., 2013), the role of the cerebellum in working memory (Davis et al., 2014) and the assumption that DBS ameliorates tremor through overriding abnormal cerebellar activity in the cerebello-cerebral circuit (Groppa et al., 2014), it is reasonable to expect an effect of stimulation not only during motor tasks, but also during cognitively demanding tasks engaging working memory.

The mechanisms of action behind the treatment effect of DBS are unknown, but cZi-DBS has been shown to modulate the activity of the sensorimotor cerebello-cerebral circuit during motor tasks (Awad et al., 2020). Whether the activity in the cerebello-cerebral circuit is modulated by DBS during tasks involving working memory is unknown. Therefore, the aim of the present study is to investigate the possible effects of cZi DBS on working-memory processing in ET patients by means of task-based blood oxygen level-dependent (BOLD) functional magnetic resonance imaging (fMRI).

## 2 Material and methods

### 2.1 Ethical considerations

The study was approved by the Umeå Regional Ethical Review Board, dnr 2011-302-31M. All patients provided written informed consent, and the study was performed in accordance with the Declaration of Helsinki 2.2.

### 2.2 Patients

Thirteen patients, eight with unilateral and five with bilateral cZi DBS, were included. The sample included 8 males, with a mean age of 68.9(SD: 8.6, range 52–78 years). Patient characteristics and stimulation parameters are presented in Table 1. The patients included in this study were initially included as part of another fMRI study focusing on DBS effects on the activity of the cerebello-thalamo-cerebral circuit in ET patients (Awad et al., 2020). All ET patients who had received cZi DBS at the DBS unit at the University Hospital of Umeå prior to the start of the study in 2015 (n = 60) were considered for participation. Thirty-five were excluded due to significant head tremor, cognitive impairment/dementia, claustrophobia, or MR-incompatible DBS system. Thus, 25 patients were asked to participate. Seventeen consented, but one died from unrelated causes before the initiation of the study, leaving 16 patients to be included. Two additional patients were excluded during the initial trials of the present study because they were unable to comprehend the instructions regarding the working memory task. Finally, behavioral and brain imaging data were excluded for one participant, as the performance in the tasks indicated that the individual had not followed the instructions/understood the task. In addition, in one participant, the first 18 vol had to be excluded due to excessive head movement in one session.

**Table 1:** Stimulation parameters and patient demographics.

patient	Age at fMRI	Comorbidity	Handedness	ETRS Off/On	DBS location	Stimulation mode	Frequency (Hz)	Voltage (V)	Pulse (μs)
**1**	75	None	L	60/26	Right	Monopolar	130	2,3	60
**2**	78	Rest tremor (No PD), disc herniation, hypertension	R	58/28	Left	Monopolar	150	2,7	60
**3**	78	Polyneuropathy. Hypothyreosis, CABG-surgery, gastric-banding surgery, COPD	R	62/21	Left	Bipolar	160	1,2	60
**5**	59	Dystonia in right hand, minor dystonia neck, hypertension	R	41/8	Bilateral	Monopolar	130	1,5	60
**6**	67	Type-II diabetes	R	41/17	Left	Monopolar	160	1,8	60
**7**	78	Polymyalgia reumatica	R	35/10	Left	Monopolar	140	1,6	60
**9**	67	Sleep apnea	R	41/20	Left	Bipolar	160	1,8	60
**10**	69	Breast cancer 2010 (mastectomy 2011, with axillary dissection followed by tremor deterioration	R	67/25	Left	Monopolar	140	1,5	60
**11**	68	CABG surgery 2010	R	35/5	Bilateral	Monopolar	140	1,6	60
**12**	70	None	R	96/19	Bilateral	Monopolar	150	1,8	60
**13**	75	Bradykinesia	R	56/27	Left	Monopolar	160	2,2	60
**14**	57	Charcot-Marie-Tooth type 1, gastric bypass 2013	R	60/8	Bilateral	Monopolar	160	2,3	60
**15**	52	Malignant hyperthermia	R	116/26	Bilateral	Bipolar	140	2,5	60

Abbreviations: *CABG*: Coronary artery bypass grafting, COPD: *Chronic obstructive pulmonary disease*, PD: Parkinson's Disease.

### 2.3 Surgical procedure

**Fig. 1:**
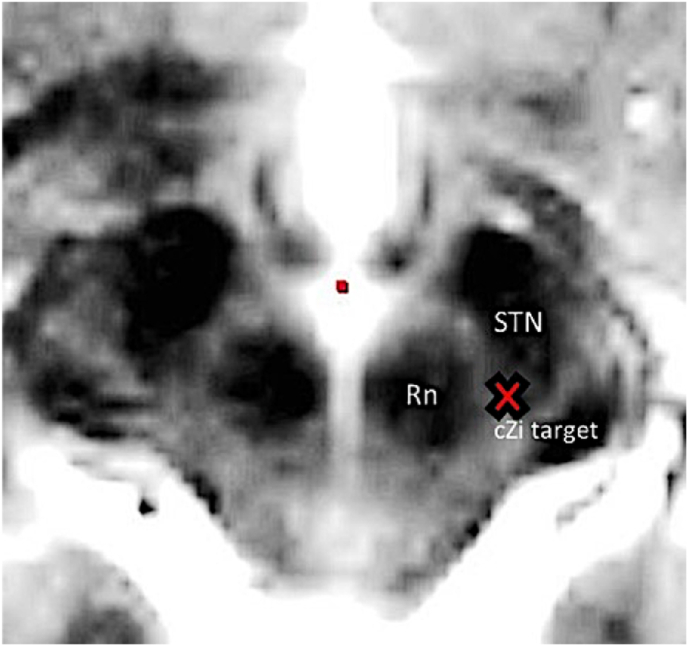
The target point in the cZi (red X), medial to the tail of the STN. Abbreviation: STN = Subthalamic Nucleus, Rn = red nucleus. (For interpretation of the references to colour in this figure legend, the reader is referred to the Web version of this article.)

The target in the cZi was visually identified on stereotactic MRI slightly posteromedial to the posterior tip of the subthalamic nucleus at the level of the maximal diameter of the red nucleus (Fig. 1). The location of the electrodes was verified using an intraoperative CT fused with the preoperative MRI. The patients were implanted with electrode model 3389 Medtronic connected to a pulse generator (Activa PC, Medtronic).

### 2.4 Experimental design

**Fig. 2:**
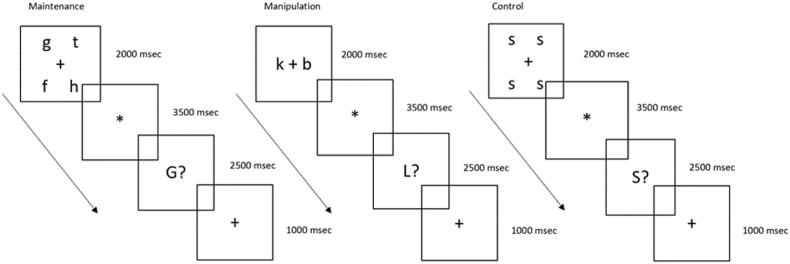
Schematic description, including the timing, of the three tasks; maintenance, manipulation, and control of the working memory task. All three examples exemplifying responses that are accurate. Abbreviations: msec = milliseconds.

The working-memory paradigm used in this study was developed by Pudas et al. (2009), originally adapted from Chee and Choo (2004). It comprises two delayed match-to-sample working-memory tasks and a control task (Fig. 2):

1. **Maintenance task**: Four target letters were shown for 2 s, followed by a fixation star. Thereafter a probe letter was shown together with a question mark. Participants were instructed to keep the four letters in memory and respond yes or no with a button press to indicate whether the probe letter was one of the four target letters.
2. **Manipulation task**: Two target letters were shown for 2 s. The participants were instructed to generate and keep the subsequent letters in the alphabet in memory. This was followed by a fixation star and a probe letter. The participants were requested to respond yes or no with a button press to indicate whether the probe letter was the subsequent letter in the alphabet to any of the two target letters.
3. **Control task**: Four identical letters were presented for 2 s, and the participants were required to only keep one letter in working memory. The participants were instructed to respond yes or no with a button press to indicate whether the probe letter was the same as the target letter.

All target letters were presented in lower case and all probe letters in capital to decrease memorization based on purely visual representation.

The tasks were presented in a blocked design, with each block consisting of three stimulus presentations of the same condition lasting for 27 s. A fixation cross was shown after each probe letter to signal a new stimulus presentation. One session consisted of 18 blocks, six repetitions each of the three tasks, presented in a pseudorandom order. An instruction screen was shown for 4 s before each new block. The procedure was repeated during two sessions for each participant, “ON” and “OFF” stimulation, with the initial ON or OFF setting counterbalanced across participants. Each session lasted about 10 min during which 188 whole-brain volumes were acquired. Stimuli were presented on a computer screen seen through a tilted double mirror and recorded by E-prime 2 (Psychology Software Tools, Inc., Pittsburgh, PA).

### 2.5 Imaging data acquisition

All scans were performed with a Philips Achieva dStream 1.5 T MR scanner using a transmit-receive (T/R) head coil, and average head-specific absorption rate (SAR) below 0.1 W/kg. Two fMRI time-series were collected per patient, one for each stimulation condition (ON and OFF stimulation). During each DBS condition (ON and OFF), three experiments/runs were collected: task-based fMRI with different motor tasks (as previously published) (Awad et al., 2020), task fMRI with a working memory task (current study) and resting state-fMRI (unpublished). The first five volumes were discarded prior to each session to allow fMRI signal equilibrium. Functional echo-planar imaging (EPI) runs were performed with the following parameters: 31 interleaved axial slices at a TR 3000 ms, TE 50 ms, voxel size 3.44 x 3.49 x 4.4 mm, 0.5 mm inter-slice gap, FOV 220 x 220 mm, and matrix size 64 x 63. An axial T1-weighted structural scan was collected after the first functional session with the following acquisition parameters: 180 slices, no inter-slice gap, 1 x 1 x 1 mm voxel size, TR 7.4 s, TE 3.4 ms, flip angle 8°, field of view (FOV) 256 × 232 mm, matrix size 256 x 232, acquisition time 11.32 min.

### 2.6 Data analyses

Behavioral data were analyzed as Accuracy and Response Time (RT). Accuracy was defined as the number of correct answers for each of the three tasks (maximum of 18). Omitted answers were considered as incorrect answers. For RT measures, individual median RTs over task repetitions were used to minimize the effect of extreme values. RTs were measured in milliseconds (ms) from onset of probe. Accuracy data of one participant was excluded due to a presumed “yes”-button malfunction during the first session, resulting in only correct rejections and misses but no hits or false alarms. The proportion of correct answers varied according to task difficulty. RT data for this participant was therefore considered representative, as there was no difference in RT between correct rejections and hits nor between false alarms and misses in the sample as a whole. Consequently, RT and brain imaging data were analyzed for 13 participants (8 male, mean age: 68.9 (SD 8.6), range: 52–78) and accuracy data for 12 participants (7 male, mean age: 68.1 (SD 8.6), range: 52–78). The effects of DBS setting (ON or OFF), and task (Maintenance, Manipulation or Control) were investigated with a repeated-measures ANOVA for each performance measure. All behavioral data were analyzed with IBM SPSS Statistics 28 and statistical significance level was set to p < .05.

FMRI data were preprocessed and analyzed with SPM12 (Wellcome Trust Centre for Neuroimaging, London, UK) implemented in Matlab R2014b. Batching of SPM routines was performed using DataZ, a software extension for SPM developed in-house. Prior to statistical analysis, the data were preprocessed with slice timing correction, movement correction with unwarping, realignment to the first image of each time series, smoothing with a 10 mm Gaussian kernel and normalized to MNI standard space. FMRI data were analyzed using a GLM (general linear model). Each of the baseline and experimental tasks (Manipulation, Maintenance, and Control) were modelled as a fixed response (boxcar) regressors of interest, convolved with the canonical hemodynamic response function. The regression model also included head-motion parameters from the realignment step, as nuisance regressors.

To optimize sensitivity in the face of the multiple-comparisons problem, and motivated by previous literature using the same task (Pudas et al., 2009) as well as the association between cerebellum and ET (Tröster et al., 2002; Benito-Leon et al., 2015), a region-of-interest (ROI) approach was applied. This approach was also motivated by suspicions that any effect on cognition from turning the DBS stimulation ON/OFF would be modest at best, and by the small sample size in the present study. The ROIs were functionally defined from voxels showing peak activations when subtracting the Manipulation task from the Maintenance task with DBS turned off.

## 3 Results

### 3.1 Behavioral data

#### 3.1.1 Accuracy

**Fig. 3a:**
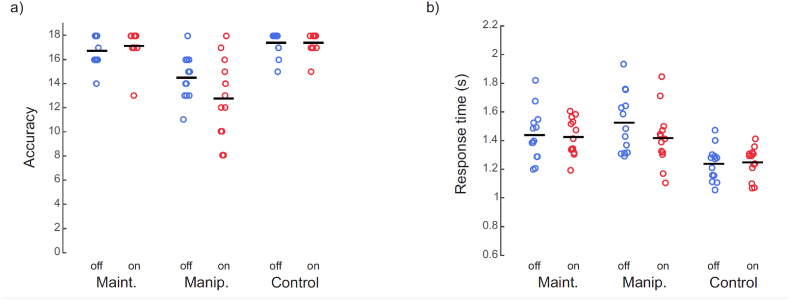
Individual plots of accurate responses in the three tasks. The horizontal bars represent group mean values (maximum 18). The main effect of task was significant (F = 26.07, **p = .000002**), with more errors in Manipulation, compared to both Maintenance and Control. **3b)** Individual plots of median response time in the three tasks, horizontal bars indicting group mean RT. The main effect of Task was significant (F = 22.102, **p = .000004**), with the fastest RTs in the control task, followed by maintenance and manipulation. Abbreviations: Maint = maintenance, Manip = manipulation.

The accuracy data for the three tasks with DBS OFF or ON are plotted in Fig. 3a. The mean accuracy (±SD) for the three tasks was: Maintenance OFF 16.8(±1.3), ON 17.2(±1.4); Manipulation OFF 14.5(±1.9), ON 12.8(±3.4); Control OFF 17.4(±1.0), ON 17.4(±0.9). A repeated-measures DBS-by-Task (2x3) ANOVA revealed no main effect of DBS setting (F = 1.77, p = .210), while the Task-x-DBS interaction was at trend level (F = 2.71, p = .089). The main effect of task was significant (F = 26.07, **p = .000002**), with more errors in Manipulation, compared to both Maintenance and Control. From the distribution of individual accuracy (see Fig. 3a), it seems that the DBS may affect performance specifically during the manipulation task, resulting in poorer accuracy with the stimulators ON than OFF. However, a direct comparison (Wilcoxon matched-pairs signed rank test) revealed only a trend (z = −1.75, p = .08). To further examine this trend, we applied the inverse efficiency score (IES) (Townsend and Ashby, 1983). A repeated-measures DBS-by-Task (2x3) ANOVA revealed no main effect of DBS setting (F = 0.318, p = .585), and no Task-x-DBS interaction (F = 0.165, p = .849).

#### 3.1.2 Reaction time

The median RT data for the three tasks with DBS OFF or ON are plotted in Fig. 3b. Group level RT for the three tasks was: Maintenance OFF: 1299.8 ms (SD ± 280), ON: 1279.0 ms (SD ± 202); Manipulation OFF: 1433.7 ms (SD ± 327), ON: 1267.9 ms (SD ± 309); Control OFF, 988.6 ms (SD ± 187), ON: 1002.3 ms (SD ± 170). A repeated-measures DBS-by-Task (2x3) ANOVA revealed no main effect of DBS (F = 1.33, p = .10), but a significant Task-x-DBS interaction (F = 3.552, **p = .045**). Post-hoc analyses of the significant Task-x-DBS interaction showed that it was due to faster RTs when DBS was switched ON relative to DBS OFF for the manipulation task (−166 ms), while the control task DBS ON generated a somewhat slower RT (+14 ms). However, these (simple) differential effects of DBS failed to reach significance (all p ≥ .1). Thus, the interaction effect was driven by inconsistencies in RT, but these differences were per se not statistically significant. The main effect of Task was significant (F = 22.102, **p = .000004**), with the fastest RTs in the control task, followed by maintenance and manipulation.

#### 3.1.3 fMRI data

The ROIs were functionally defined as regions more involved in the Manipulation task as compared to the Maintenance task (calculated from a one sample *t*-test by contrasting the Manipulation task with the Maintenance task with DBS OFF, contrast at p < .05 FWE corrected at the cluster level, cluster-defining threshold: p < .001). This resulted in two regions in the left frontal cortex and nothing else. We then relaxed the threshold to p < .001 uncorrected and chose the clusters with the peak t-value in parietal cortex and cerebellum. Within these four clusters, we averaged the beta values across voxels and imported them to SPSS for further analysis, using a 3x2 repeated-measures ANOVA. A p-value of < .05 was considered significant. Thus, performing the manipulation task relative to maintenance, regardless of DBS stimulation setting, was associated with an expected BOLD signal increase in the DLPFC, MFG, parietal area and cerebellum (see Table 2 for coordinates).

**Table 2:** Clusters with higher BOLD signal during Manipulation compared to Maintenance.

Anatomical Location	x	y	z
**Frontal**			
DLPFC	−40	6	38
MFG	−34	54	−2
**Parietal**			
Left Angular Gyrus	−36	−54	34
**Cerebellum**			
Right Crus II	14	−88	−38

Abbreviations: DLPFC: Dorsolateral Prefrontal Cortex; MFG: Middle Frontal Gyrus; x, y, z coordinates in MNI (Montreal Neurological Institute) stereotaxic space.

**Fig. 4:**
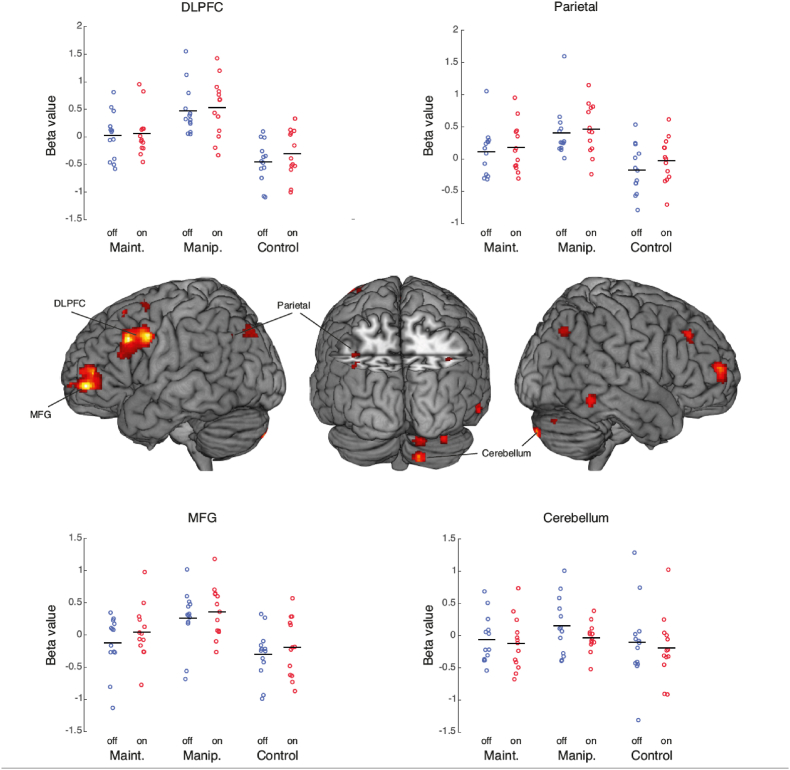
BOLD signal change in the DLPFC, MFG, parietal cortex, and cerebellum for the three tasks (plots). Solid line = average signal change. The brain rendering is made at p < .001 uncorrected for illustration, see main text for ROI definition criteria. Abbreviations: Manip. = manipulation, Maint. = maintenance, DLPFC = dorsolateral prefrontal cortex, MFG = middle frontal gyrus.

A 3x2 repeated-measures ANOVA revealed a significant main effect of task in three of the chosen ROIs (see Fig. 4), with increasing activation from Control to Maintenance to Manipulation, which activated the areas the most. DLPFC: F = 41.53, **p = 1.6x10**^**−8**^; MFG: F = 32.32, **p = 1.5x10**^**−7**^; Parietal: F = 29.12, **p = 3.8x10**^**−7**^, Cerebellar: F = 3.31, p = .054. There were no significant effects of DBS ON/OFF (DLPFC: F = 0.29, p = .60, MFG: F = 0.49, p = .49, Parietal: F = 0.33, p = .58, Cerebellar: F = 0.60, p = .45), or Task-x-DBS interaction (on/off) (DLPFC: F = 0.57, p = .57; MFG: F = 0.60, p = .56, Parietal: F = 1.30, p = .29; Cerebellar: F = 1.08, p = .36).

The ROI approach comes with the cost of not testing BOLD signal change in all brain regions, and a conclusion that there are no BOLD signal alterations from DBS stimulation based on a ROI analysis alone could therefore be seen as misleading. Therefore, we also did a whole-brain analyses investigating the main effect of DBS (DBS ON/OFF) and the Task-x-DBS interaction. No changes in BOLD signal were significant (p = .05 FWE corrected at the cluster level, cluster-defining threshold = 0.001).

## 4 Discussion

The aim of the present study was to investigate the possible effects of cZi DBS on working-memory processing in ET patients by means of task-based BOLD fMRI. The effects of cZi DBS in patients with ET performing a working memory task on the cerebello-thalamo-cerebral circuit has, to our knowledge, not previously been explored by functional neuroimaging. The results revealed no significant effects of cZi DBS, indicating that stimulation in the cZi does not affect brain activity in patients with ET when completing a working-memory task.

The DLPFC has long been suggested to be important in response selection through inhibition of competing and inappropriate responses (Jahanshahi et al., 2000; Rowe and Passingham, 2001). The fronto-parietal cortical regions are also thought to constitute a core circuit in working-memory maintenance and are involved in processing components such as maintaining goals and task sets (Eriksson et al., 2015). Previous fMRI studies have used similar working-memory tasks to investigate possible effects of a variety of cognitive disadvantages, such as aging (Rieckmann et al., 2017) and genetic variances combined with aging (Nyberg et al., 2014) on brain activity and working memory. The results have indicated that individuals with a cognitive disadvantage recruit the DLPFC and parietal cortex areas maximally already during simpler maintenance tasks, which prevents them from reaching higher brain activation levels during the more demanding manipulation task, i.e., less cognitive efficiency. WM decline after the age of 50 and a weaker DLPFC response during manipulation, together with a stronger response during maintenance is also common in older adults (Nyberg et al., 2012).

The present study revealed a significant Task-x-DBS interaction, with faster RTs during DBS ON relative to DBS OFF for the manipulation task. However, post hoc tests of condition-specific effects of DBS ON RTs failed to reach significance. The significant effect on RT with DBS ON could be interpreted as a positive effect of DBS on certain cognitive functions (psychomotor speed, vigilance, sustained attention), but combined with an increased error rate in Manipulation, it could also indicate a negative trade off on response selection and inhibition. There was a non-significant trend towards an increased error rate during DBS ON in the manipulation task, which combined with the faster RTs with DBS ON in this task, suggested a trend towards a speed-accuracy tradeoff. As part of our post-hoc analysis we examined this trend by applying the inverse efficiency score, but the results were non-significant. Stimulation-induced modulations of speed-accuracy tradeoffs have been documented for STN-DBS in Parkinson's disease when patients act under speed pressure (Pote et al., 2016). These behavioral trends, albeit non-significant, would be of interest to analyze in a larger sample. Another aspect that would be of interest to investigate further would be to look at individual differences, for example to determine whether individuals with relatively impaired working memory preoperatively experience more pronounced alterations during postoperative DBS ON. Conducting such correlation analyses would however require a larger sample size.

Functional brain abnormalities in patients with ET have been reported in several fMRI studies. These include abnormalities in the cerebello-thalamo-cerebral network, as well as increased connectivity in resting-state networks involved in cognitive processes (Wang et al., 2018). Compared to healthy controls, ET patients have been found to exhibit *increased* resting-state functional connectivity in regions involved in cognitive processes (default mode network and fronto-parietal network) and *decreased* functional connectivity in the cerebellum and visual networks (Benito-Leon et al., 2015). These changes were associated not only with the severity and duration of the disease, but also with cognitive ability. Increased connectivity in the default mode network and fronto-parietal network was associated with worse performance on different cognitive domains and depressive symptoms. However, decreased connectivity in the visual network, was associated with worse performance on visuospatial ability. Graph theory analysis has also been used to assess functional network organization (Benito‐León et al., 2019). The results indicated that the efficiency of overall brain functional networks in ET was disrupted. This would then support the idea that ET disrupts widespread brain regions, including those outside of brain regions primarily responsible for tremor generation.

Literally meaning “the zone of uncertainty”, the Zona Incerta (Zi) is a neuroanatomically diverse, elongated nucleus in the diencephalon roughly between the subthalamic nucleus, the medial lemniscus, and the red nucleus (Fytagoridis et al., 2012; Mitrofanis, 2005). While its caudal part seems to play a role in the motor system, other areas are associated with functions such as arousal, attention, and visceral control (Mitrofanis, 2005). The different subsectors of the Zi communicate with each other both within and across hemispheres. Thus, stimulation of the Zi can influence various aspects of brain activity in both hemispheres.

The aim of this study was to evaluate to the possible effects of cZi DBS on working-memory processing in ET in ON-OFF conditions. A previous study (Philipson et al., 2019) focusing on cognitive outcome 12 months following Zi-DBS in patients with ET did not reveal any major negative effects on the tests of cognitive function included, other than a slight decline of semantic verbal fluency. Thus, the present imaging results, together with the results of the aforementioned study (Philipson et al., 2019), suggest that the effects of such stimulation on cognitive function are marginal. This is of interest from a clinical perspective and indicate that DBS in the Zi in patients with ET does not produce WM alterations over time.

Our study has several limitations, such as a limited sample of only 13 patients and lack of control group. The results should therefore be interpreted with caution. A limited sample size will of course require that our results are confirmed in future studies, preferably with larger sample sizes. However, this is the first study investigating possible effects of cZi DBS on the cerebello-cerebral circuit in patients with ET. The fact that no significant changes were found is in itself interesting and may support the claim that the cZi is a safe target with regards to cognitive side-effects when treating patients with ET. Despite the limited sample size, we believe that our results are of interest and hope that our study will encourage others to verify or dispute our findings.

## 5 Conclusions

The present study indicates that DBS in the cZi in patients with ET at most marginally affects working memory. From a clinical point of view, it is important to identify any unwanted side-effects, including those of a cognitive nature, when evaluating a novel target. Thus, the lack of adverse cognitive effects documented in pre-vs post-operative neuropsychological evaluation of ET patients with cZi DBS (Philipson et al., 2019) and the results of the present imaging study, is important when evaluating DBS in the cZi as a safe and effective alternative in DBS treatment of ET patients.

## CRediT authorship contribution statement

**Johanna Philipson:** Formal analysis, Writing – original draft, Writing – review & editing. **Amar Awad:** Conceptualization, Methodology, Investigation, Writing – review & editing. **Lena Lindström:** Formal analysis, Writing – original draft, Writing – review & editing. **Patric Blomstedt:** Conceptualization, Methodology, Resources, Writing – review & editing. **Marjan Jahanshahi:** Supervision, Writing – review & editing. **Johan Eriksson:** Conceptualization, Methodology, Formal analysis, Project administration, Writing – review & editing, Supervision.

## Declaration of competing interest

The authors declare the following financial interests/personal relationships which may be considered as potential competing interests: Patric Blomstedt is consultant for Abbott and Boston Scientific and shareholder in Mithridaticum AB. He has also been awarded a grant from Vetenskapsrådet.

## Data Availability

Data will be made available on request.
